# What Is the Essence of Microbial Electroactivity?

**DOI:** 10.3389/fmicb.2016.01890

**Published:** 2016-11-25

**Authors:** Christin Koch, Falk Harnisch

**Affiliations:** Department of Environmental Microbiology, UFZ-Helmholtz Centre for Environmental ResearchLeipzig, Germany

**Keywords:** extracellular electron transfer, exoelectrogenic, syntrophy, microbial electrochemical technology, *Geobacter*, microbial fuel cell

Microorganisms performing extracellular electron transfer (EET) show electroactivity and are the fundament of primary microbial electrochemical technologies (MET) (Schröder et al., 2015) as well as key players of geochemical cycles (Newman and Banfield, 2002; Melton et al., 2014). However, only a few electroactive microorganisms, like *Geobacter* or *Shewanella*, are studied in detail, e.g., for their electron transfer mechanisms (Gorby et al., 2006; Brutinel and Gralnick, 2012; Lovley, 2012). Many more species are only globally assigned to be electroactive (Koch and Harnisch, 2016), but mechanistic knowledge is generally missing and the natural importance of this trait not comprehensively understood.

However, there is no common definition of electroactivity and a genetic or metabolic marker or even a gold standard does not exist. This lack together with the high diversity of electroactive microorganisms—with regard to their phylogeny but also their physiology—challenges a systematic assessment and comparison (Koch and Harnisch, 2016). This difficulty is furthermore accelerated by the diversity of experimental setups and techniques exploited (Harnisch and Rabaey, 2012). The deficit of a stringent definition of electroactivity may sound purely academic from an application or engineering perspective. However, it is not. A consensus on electroactivity combined with good craftsmanship (Egli, 2015) for studying and engineering electroactive microorganisms as well as MET has to form the fundament of future research and development. The following treatise is certainly not comprehensive, but we will show that a better understanding of the linkage between EET, microbial metabolism, and system performance is necessary to form this fundament or in other words “To distil the essence of electroactivity.”

## Agony of choice or how would you decide?

**Figure 1:**
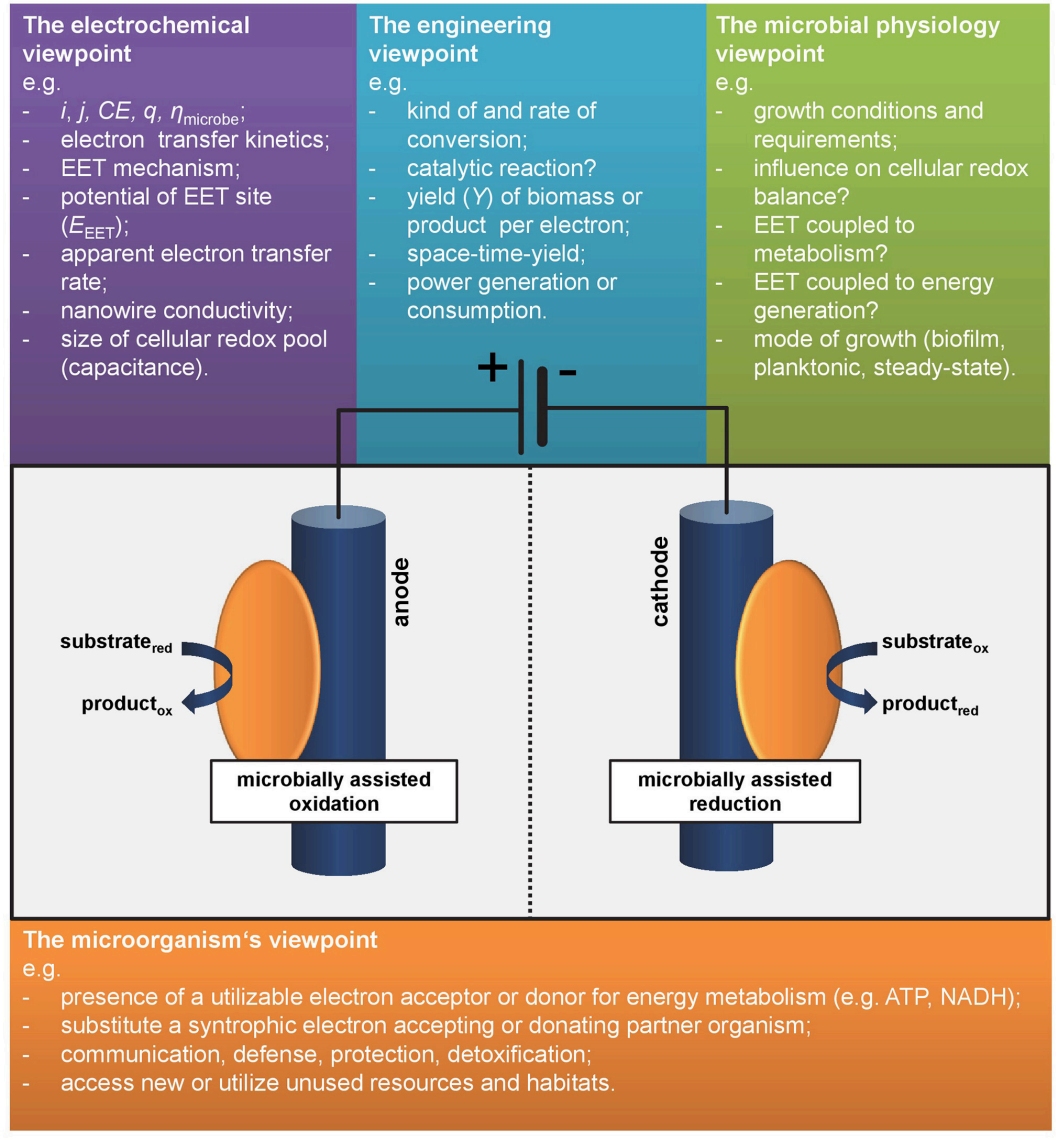
**Different viewpoints on electroactivity**. As there is no common definition or marker defining species as electroactive, different disciplines have different viewpoints.

Considering two electroactive microbes A and B, which one can be defined to be more electroactive? Microbe A being psychrophlic and performing (slow) EET (hence low current density^1^ *j*) at 10°C with a coulombic efficiency^2^ (*CE*) close to 100%—or—microbe B being thermophilic and performing fast EET (hence high *j*) at 60°C with low *CE*? The decision is not straightforward and would usually depend on the respective process as well as *j* and *CE* required or feasible for its application. Interestingly this example illustrates the common sense in the perception of electroactivity of microorganisms. In the outmost majority of studies system level parameters are used for characterization. These numbers related to the engineering or electrochemistry viewpoint are (i) the overall yield of electrons from a substrate (at anodes) or stored in a product (for cathodes) as expressed in *CE* and (ii) the “speed” of EET, i.e., electrons per time unit usually expressed as current, *i*, or normalized to electrode surface area respectively volume as current density, *j* (Harnisch and Rabaey, 2012; Schröder et al., 2015). Noteworthy, these performance parameters can be, but not have to be linked to the metabolic level of microbes A and B as will be discussed in the following section and hence are not generally suitable for assessing what electroactivity is. As shown below, the answer to the introductorily question will strongly depend on the individual perspective (see also Figure 1).

## What are typical characteristics that define microorganisms as electroactive (or not)?

Let us consider our model organism *Geobacter sulfurreducens* and its relatives: they form biofilms at anodes while oxidizing acetate and performing direct electron transfer and most express conductive nanowires. Under anodic growth conditions with the electrode as only electron acceptor the microbial metabolism is completely dependent on the EET as this is the only pathway of energy generation. This species can be clearly defined as electroactive. But how to compare it to other species differing from this model organism? Choosing an adequate measure is difficult as summarized by the different viewpoints on electroactivity in Figure 1.

## From microbial cells to electrochemical cells and back again

For microorganisms forming biofilms at electrodes like *Geobacter sulfurreducens*, the biofilm thickness^3^ might seem as a measure of electroactivity, as a higher cell number might go along with an increased current flow. However, differences in cell density and biofilm thickness can be specific for microbial species (and already obvious differences exist within the family of *Geobacteraceae*), thereby being dependent on its (local) environment, growth state etc. (Bonanni et al., 2013; Tan et al., 2016). From a practical perspective measuring typical parameters like, e.g., cell number, dry weight, etc. of biofilms is not simple and mostly destructive for the object of study. Hence, time resolved analyses are challenging, especially when considering what a “representative” sample is. For mixed culture biofilms it was shown that shear stress can effect the biofilm thickness and the biomass density (Pham et al., 2008). This can lead to differences regarding the substrate turnover and electron flow for an individual cell which is not necessarily reflected by the anodic current density. In this case, the coulombic efficiency might seem a good objective measure of electroactivity being independent of cell number and also considering potential electron losses within the cell. Experimentally, even harder to determine would be the electron transfer per single cell (Liu et al., 2010; Jiang et al., 2013; Gross and El-Naggar, 2015), which from our perspective could be considered an excellent measure. On the technical scale, biomass respective number of cells, *N*_cell_, or formed product, *P*, is often in focus. Here in analogy to established parameters in biotechnology (Doran, 1995) we propose that yields per electron (e.g., cell yield per electron, *Y*_*Ne*−_, or product yield per electron, *Y*_*Pe*−_) can be defined based on the number of transferred electrons *n*_*e*−_. This number is derived from the transferred charge, *q*, and the Faraday constant, *F*.

$$Y_{Ne-}=\frac{\left(N_{cell,t}-N_{cell,0}\right)}{\left(n_{e-,t}-n_{e-,0}\right)},\text{with}\underset{0}{\int^{t}}\;idt=q\text{ and }n_{e-,t}=\;\frac{q}{F};$$

as *n*_*e*−, 0_ = 0 it follows:

$$\begin{array}{l} Y_{Ne-}=\frac{\left(N_{cell,t}-N_{cell,0}\right)}{q}\;\times F \end{array}$$

analogously it follows:

$$\begin{array}{l} Y_{Pe-}=\frac{\left(P_{t}-P_{0}\right)}{q}\;\;\times \;F,\text{when considerin}\;P_{0}=0,\\ \text{it}\;\text{simplifies to }Y_{Pe-}=\frac{P_{t\;}}{q}\;\;\times \;F. \end{array}$$

From the electrochemical viewpoint the microbial overpotential, η_microbe,_ can be defined similar to the electrochemical overpotential (Bard et al., 2012) and can be used for a thermodynamic comparison of electroactive microorganisms. For microbial anodes it is the difference between the formal potential of the substrate, *E*_substrate,_ and the formal potential of the EET site, *E*_EET_, i.e., the potential at which the electrons are released by the microbe, with η_microbe_ = *E*_substrate_− *E*_EET_. However, η_microbe_ sets only the upper limit of the microbial energy gain. Yet, the true energy gain, i.e., being stored for instance in ATP or reduction equivalents and subsequently used for anabolic reactions, is decreased due to losses, like heat dissipation (Korth et al., 2016). Here species specific differences can be expected or in other words: Is a microbe more electroactive performing fast EET that strives for its living or one that harvests a lot of energy per electron but at a very slow rate?

Another potential measure of electroactivity, so far described for *G. sulfurreducens* but easily transferable to other species, is the capacity for electron storage in the cell (Esteve-Núñez et al., 2008; Malvankar et al., 2012). This capacitor principle reflects the size of the cellular redox pool to store electrons. Compared to the above described possible measures of electroactivity on the cellular level also the subcellular level can be considered, as e.g., by measuring the apparent electron transfer rate of, e.g., cytochromes (Bonanni et al., 2012), or the conductivity of, e.g., nanowires (Adhikari et al., 2016). While the presence of cellular appendices is not sufficient for claiming a microbe as being electroactive the exact determination of the specific conductivity could prove the EET capacity and also differences in transfer efficiency and transfer kinetics could potentially be explained. So far there are only a few studies on nanowire conductivity and yet no threshold value is available.

When considering full or partly planktonic living cultures, e.g., the well-studied *Shewanellaceae*, the definition of a measure of electroactivity becomes not simpler either. Here, cell density and inhomogeneities and gradients of substrates, metabolites or mediators as well as access to the electrode surface play an additional role that makes systematic comparison even more complex (Borole et al., 2011; Harnisch and Rabaey, 2012; Patil et al., 2015).

These few considerations and surely not comprehensive treatise already shows that even for the model organisms of *Geoabacteraceae* and *Shewanellaceae* there is no straightforward measure of electroactivity.

## The diversity of electroactive livings

When considering less systematically studied microorganisms differing from the model organisms the definition of electroactivity becomes even more challenging. Up to now 94 microbial species are assigned to be electroactive and presumably significantly more electroactive species exist in nature (Koch and Harnisch, 2016). There is strong evidence that some electroactive microbes can only exist in microbial consortia. Therefore, the questions arises what makes the cells forming electroactive biofilms? Is it the sole presence of a potential electron acceptor or donor, as described for 54 electroactive pure cultures. In these cases, the electrode might serve as substitute to a syntrophic electron accepting or donating partner organism in a natural setting. But also in nature, microorganisms can be selective and not match with every potential partner organism. There is hardly anything known, which kind of communication, recognition or additional metabolite transfer might take place between the partners involved in consortia. These additional signals will not be provided by a sole electrode and therefore microorganisms performing EET, but not on electrodes, might accordingly not be identified as electroactive, but shouldn't they?

The comparison of electroactive microorganisms becomes even more vague when we consider electroactive microorganisms as all microorganisms that are able to lead to a Faradaic current flow at electrodes. This current flow can result from the connection of the electron flow to the microbial metabolism but also from a solely catalytic reaction performed within the cells, but being independent from their growth, maintenance or even metabolism. Interestingly, recently also ionic currents have shown to play an important role in microbial communities, especially their communication (Prindle et al., 2015). Further, even current flow resulting obviously from cell burst can be found in literature claiming species as electroactive. Here we disagree on their inclusion. Considering microbial electroactivity in a catalytic sense, i.e., based on chemical transformations taking place independently from the metabolism, seems a very artificial approach from a microbial physiology viewpoint and far away from any natural significance. At the same time these transformations can be considered as highly promising from the technical perspective, e.g., in bioelectrosynthesis. This also holds true for the concept of steering the microbial metabolism by interfering in the cellular redox balance, e.g., by redox mediators, in case the microorganisms do not interact with electrodes naturally (Park and Zeikus, 2000). This is nothing we observe in nature or that we could explain by its natural relevance, but still it results in an obvious wiring of microbial metabolism and electron flow. Is this microbial electroactivity?

There are plenty of examples in nature where microorganisms communicate, fight for resources and invade new habitats by producing redox active compounds like e.g., phenazines in *Pseudomonas* spp. (Price-Whelan et al., 2006). In this case, the primary aim (from the viewpoint of the microbial cell) is to communicate with other cells of the own species (quorum sensing), to defend a habitat against competitors, detoxify toxic compounds or access new resources. However, the same chemicals can also serve as redox mediators for mediated electron transfer (Rabaey et al., 2005). In these cases, the EET might be no or only a minor mode of energy generation but will rather consume additional resources and provide the involved species a short time but possibly significant selection advantage. At the same time, we can utilize these microbial capacities in technical systems but do we define these species then also as electroactive? And how can one trigger these specific activities and utilize these microorganisms long term in technical systems? Is it “healthy” for a microbial cell when interfering with their metabolic pathways using electrodes?

## On the future distilling of the essence of microbial electroactivity

Apart from maybe a dozen model organisms, the mechanism of EET in microorganisms being assigned electroactive is almost not investigated. For instance we hardly know anything about the potential electron uptake mechanism from cathodes or how gram-positive bacteria perform EET. Accordingly, it is a challenge to assess or even compare the electroactivity of different species with each other as their “motivation” for EET might be completely different as well as its connection to the cellular metabolism. Even if there is a basic understanding of the involved mechanisms it seems not applicable to compare a *CE* of anodic acetate oxidation to a cathodic nitrate reduction. We believe that the first steps for approaching a common sense will involve the definition of a set of basic microbial characteristics to be reported and experimental setups to characterize microbial electroactivity in pure cultures. It is not sufficient to “just” measure a current in the presence of microorganisms (even with sufficient replicates) to call this microbe electroactive. In addition to assessment of functional parameters (e.g., current density, *CE*) in a standardized setup the understanding of the functional connection of current flow and microbial metabolism should be aimed. As detailed above this functional characterization is not straightforward for pure cultures yet, and represents an even greater challenge when mixed cultures are considered.

Finally, a community-wide discussion leading to a (even preliminary) common sense of electroactivity is needed. Even then there seems to be not only one kind of electroactivity and assigning a microbe electroactive or not might be in the eye of the beholder or in other words “It is the distiller's personal finest selection.”

## Author contributions

All authors listed, have made substantial, direct and intellectual contribution to the work, and approved it for publication.

### Conflict of interest statement

The authors declare that the research was conducted in the absence of any commercial or financial relationships that could be construed as a potential conflict of interest.

## Footnotes

^1^The current, *i* (usually in mA) represents the number of the transferred electrons per second to an electrode and hence can be a measure of the metabolic activity. For normalization often the current density is used by relating the current to the (geometric) surface area of an electrode, *j*_geo_ (in mA cm^−2^), or to the volume of the electrode chamber, *j*_vol_(in mA cm^−3^).

^2^The coulombic efficiency, *CE*, is a measure of the ratio of theoretical available electrons from the substrate and the transferred electrons to the electrode at the anode; hence it can be regarded as measure of metabolic efficiency.

^3^Here we refer to (i) the thickness in steady-state conditions and (ii) to uniform biofilms. The definition of steady state and its experimental realization as well as biofilm stratification are worth its own treatise.
